# Vitamin A supplementation reduces the Th17-Treg – Related cytokines in obese and non-obese women

**DOI:** 10.1590/2359-3997000000125

**Published:** 2016-01-01

**Authors:** Mahdieh Abbasalizad Farhangi, Ali Akbar Saboor-Yaraghi, Seyyed Ali Keshavarz

**Affiliations:** 1 Department of Community Nutrition School of Nutrition Tabriz University of Medical Sciences Tabriz Iran Nutrition Research Center, Department of Community Nutrition, School of Nutrition, Tabriz University of Medical Sciences, Tabriz, Iran; 2 Department of Cellular and Molecular Nutrition School of Nutrition and Dietetics Tehran University of Medical Sciences Tehran Iran Department of Cellular and Molecular Nutrition, School of Nutrition and Dietetics, Tehran University of Medical Sciences, Tehran, Iran; 3 Department of Clinical Nutrition School of Nutrition and Dietetics Tehran University of Medical Sciences Tehran Iran Department of Clinical Nutrition, School of Nutrition and Dietetics, Tehran University of Medical Sciences, Tehran, Iran

**Keywords:** Vitamin A, obesity, Th17, Treg, inflammation

## Abstract

**Objective:**

The objective of the present study was to investigate the effect of vitamin A supplementation on serum Th17 (IL-6, IL-17, IFNγ) and Treg (TGF-β, IL-10) related cytokines in obese and non-obese women.

**Subjects and methods:**

In a randomized double blind placebo controlled design, 56 obese women were randomly assigned to receive either an oral dose of 25,000 IU retinyl palmitate or placebo per day for 4 months. Twenty eight ages matched non-obese women were also received vitamin A. At the study entry, anthropometric variables were measured and serum Th17 and Treg related cytokine profile were determined at baseline and 4 months after intervention.

**Results:**

Significantly higher baseline concentrations of IL-6 were observed in obese compared with non-obese women (P < 0.05). However, the initial concentrations of other cytokines were not significantly different between groups. The mean concentrations of IL-17 and TGF-β were significantly decreased after vitamin A supplementation in non-obese and obese women respectively. Positive relationships between IL-17 and IL-10 (r = 0.42, P < 0.001), TGF-β and IL-17 (r = 0.35, P < 0.001) and between IL-10 and IFN-γ (r = 0.41, P = 0.002) in total participants were also observed.

**Conclusions:**

The results of the present study showed for the first time that vitamin A supplementation reduces serum concentrations of IL-17 and TGF-β in reproductive age women. Further studies are needed to explore the possible underlying mechanisms.

## INTRODUCTION

Obesity, the modern chronic disease of twenty-first century, is rapidly increasing worldwide particularly in developing countries. This high prevalence of obesity is considered as a major health concern due to close relationship between obesity and numerous metabolic abnormalities and chronic disease including dyslipidemia, insulin resistance, hypertension, cardiovascular disease, several types of cancers and autoimmune disease (1,2). A large body of evidence suggests that many of these obesity associated co-morbidities have a long term low grade inflammatory basis (3). Among inflammatory biomarkers, the role of classic inflammatory mediators including tumor necrosis factor α (TNF-α) and C-reactive protein (CRP) have been extensively investigated in obesity (4,5). However, recent studies have revealed the possible role of Th17-T cell sub-lineage in obesity associated metabolic and autoimmune disorders. Winer and cols. (6) have demonstrated that diet induced obese mice have elevated Th17-T cell pools and produce more IL-17 compared with lean mice. Additionally, the elevated Th17 cells in obese mice was associated with progressively enhanced incidence of two models of autoimmune disease including experimental autoimmune encephalomyelitis** (**EAE) and trinitrobenzene sulfonic acid colitis. In another study by Sumarac-Dumanovic and cols. (7) obesity was associated with higher blood concentrations of IL-17/IL-23 axis. IL-17 is a potent pleiotropic pro-inflammatory cytokine that participates in tissue inflammation by activating several cytokines, matrix metalloproteases and stimulating neutrophils proliferation and migration and its concentrations is increased in sera and tissue of several autoimmune disease such as rheumatoid arthritis, multiple sclerosis and inflammatory bowel disease (8,9). On the other hand, regulatory T-cells (Treg) have anti-inflammatory effect and can suppress autoimmunity by releasing anti- inflammatory cytokines (TGF-β and IL-10) (10). Vitamin A and its metabolite, all trans retinoic acid (ATRA) are known to regulate immunity and ameliorate several autoimmune disease in animal models (11) however, the role of vitamin A in production of Th17/Treg cytokines in obese individuals has not been defined. Therefore, we explore the effect of vitamin A supplementation on serum Th17/Treg related cytokines in obese women.

## SUBJECTS AND METHODS

### Study population

This study included 84 women who fifty six were obese (BMI 30-35 kg/m^2^) and twenty eight were non obese (BMI 18.5-24.9 kg/m^2^). The subjects were healthy volunteers and were invited to participate in the study through invitation letters. Inclusion criteria were as follows: age 20-52 years, absence of diabetes, thyroid abnormalities, chronic liver or renal disease, autoimmune disease and any other disease that might affect immune function, no consumption of any dietary vitamin A supplements or no treatment with drugs that may interfere with absorption or bioavailability of the supplement. The study protocol was approved by ethics committee of Tehran University of Medical Sciences (TUMS) and written full-informed consent was obtained from all of subjects before participation in the study. This trial was also registered at Clinicaltrials.gov (Identifier NCT-01405352).

### Experimental protocol

Obese women were divided into two groups using random permuted block randomization. One group received an oral dose of vitamin A as 25,000 IU/d retinyl palmitate [~7576 retinol equivalent (RE)/d] and other group received placebo during 120 days. The non-obese group, who was matched with obese groups for age, also received vitamin A in the same dose. The intervention period was 120 days and subjects were followed by monthly phone contacts. Vitamin and placebo softgels were obtained by Zahravi Pharmaceutical Inc. (Tabriz-Iran) and were identical in appearances and color. Selecting the dose of 25,000 IU for vitamin A supplements was based on the previous studies (12) supporting that vitamin A in this dose can increase circulating concentrations of all trans retinoic acid and 9-cis retinoic acid to concentrations that will interact with their respective receptors (RAR and RXR). The participants were advised to have their usual diet and physical activity and to avoid pregnancy during the study period. All of subjects underwent full anthropometric measurements including body weight, height, waist circumference and hip circumference at the study entry. Weight was measured by calibrated Seca scale (Itin Scale Co., Inc. Germany) with the precision of 0.1 and height measurement had taken by a cotton ruler. BMI was calculated as weight (kg)/(height (m))^2^. The waist circumference (WC) was taken above the iliac crest at the natural waistline and the hip circumference (HC) was taken at the largest area of the natural hip line. Waist to hip ratio (WHR) was calculated by dividing values of waist (cm) to hip (cm). Physical activity was obtained by international physical activity questionnaire (IPAQ).

### Blood collection and IL- 6, IL-17, TGF-β and IFN-γ analysis

Venous blood samples were collected in the morning after an overnight fasting at the beginning of the study and after four months. The blood samples were collected in tubes with no additives. These samples were centrifuged; the serum was obtained and aliquots immediately stored at -70ºC until assay. Serum cytokines were measured with commercial ELISA kits (e-Bioscience, San Diego, CA, USA). The assays were on a dual–antibody sandwich principle. Each assay was performed with recombinant cytokine using 96-well micro plates (Costar 9018; Corning Costar Corp., Corning, NY, USA). Briefly, the cytokine assay procedure comprises from following stages: coating with capture antibody, overnight incubation at 4°C, blockage the plates with assay diluents, adding the diluted standard solutions and sera to appropriate wells, incubation at 37°C for 2 hours, adding detection antibody (Biotin conjugate anti-human cytokine) and avidin–Horseradish Peroxidase (HRP). Finally, adding Tetramethylbenzidine (TMB) as substrate solution and Sulfuric acid 2N as stop solution. For TGF-β analysis a prior acid activation of serum samples with HCl (1N) is also needed to activate latent TGF-β1 to its immunoreactive form.

Sensitivities of assays were as follows: 2 pg/mL (IL-6), 2 pg/mL (IL-10), 4 pg/mL (IL-17), 60 pg /mL (TGF-β) and 4 pg/mL (IFN-γ). Inter and intra-assay coefficients of variation were < 10%.

### Statistics

Kolmogorov-Smirnov test was applied to test normality of distribution. Comparison of variables between groups was performed by one way ANOVA for parametric tests using a Tukey’s *post-hoc* test for multiple comparisons. Comparison of non – parametric data was performed with Kruskal Wallis test using a Bonferroni *post-hoc* test for multiple comparisons. Paired *t*-test or Wilcoxon- signed rank test was performed to determine the effect of intervention on biochemical variables. Adjustments for the difference in baseline values of variables were performed by ANCOVA using general linear models. Correlation between variables was analyzed by Pearson or Spearman rank correlation tests. All statistics were two – tailed and a *P* value less than 0.05 was considered significant. Data processing and statistics were performed by SPSS software (version 11.5, SPSS Inc., Chicago, IL, USA).

## RESULTS

During the intervention period, a total of nine women (10.71%) withdrew from the study. Six withdrawals were in obese groups (3, irregular use of supplement or placebo, 2, impossibility to be present at appointment and 1, unwillingness to continue the trial). Three withdrawals were in non obese group (1, pregnancy and 2 irregular use of supplement). A total of 75 women completed the study. General characteristics of study participants are presented in Table 1. Weight, BMI, WC, HC and WHR in obese groups were significantly higher than non-obese group. Mean age and physical activity score were not different between groups. The results of dietary intake of energy and nutrients and also change in BMI and other anthropometric variables have been reported before (13). There were no significant differences between dietary intakes of energy, carbohydrate, protein, fat, vitamin A and beta-carotene between treatment groups. No change in weight, waist circumference and BMI has been observed in study groups after intervention (13). Serum cytokines were not in significant relationships with none of anthropometric parameters (data not shown).

**Table 1:** Baseline characteristics of trial participants

	Obese – vitamin A	Obese – placebo	Non-obese	P*
n	27	23	25	
Age (years)	38.57 ± 1.32	38.92 ± 1.60	36.03 ± 1.37	0.31
Weight (kg)	82.77 ± 1.52	79.03 ± 1.87	58.40 ± 1.36	< 0.001
BMI (kg/m^2^)	33.74 ± 0.64	31.50 ± 0.72	22.80 ± 0.40	< 0.001
WC (cm)	95.64 ± 1.31	93.51 ± 1.47	75.96 ± 1.25	< 0.001
HC (cm)	115.35 ± 1.57	112.21 ± 1.64	99.10 ± 1.24	< 0.001
WHR	0.82 ± 0.004	0.83 ± 0.007	0.76 ± 0.005	< 0.001
PA (Met-min/week)	2225.50 ± 234.06	2287.26 ± 534.40	2594.33 ± 251.10	0.11

BMI: body mass index; WC: waist circumference; HC: hip circumference; WHR: waist to hip ratio; PA: physical activity.

* Obese groups have significantly higher levels than non-obese group (multiple comparisons using Tukey’s post-hoc test).

**Figure 1:**
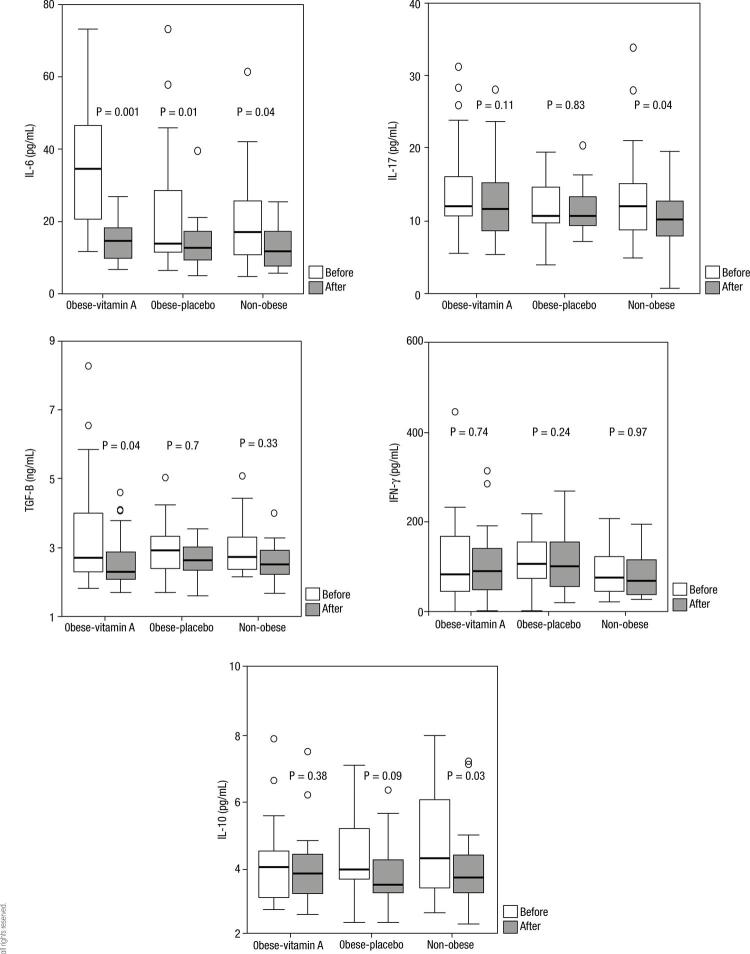
Serum concentrations of Th17 and Treg related cytokines in obese and non-obese groups before and after intervention in box plots; data are shown as median values (horizontal lines), interquartile ranges (the box lengths), extreme values (whiskers) and outliers (o). P values are for comparisons of variables before and after intervention and are determined by Wilcoxon signed rank test).

**Figure 2:**
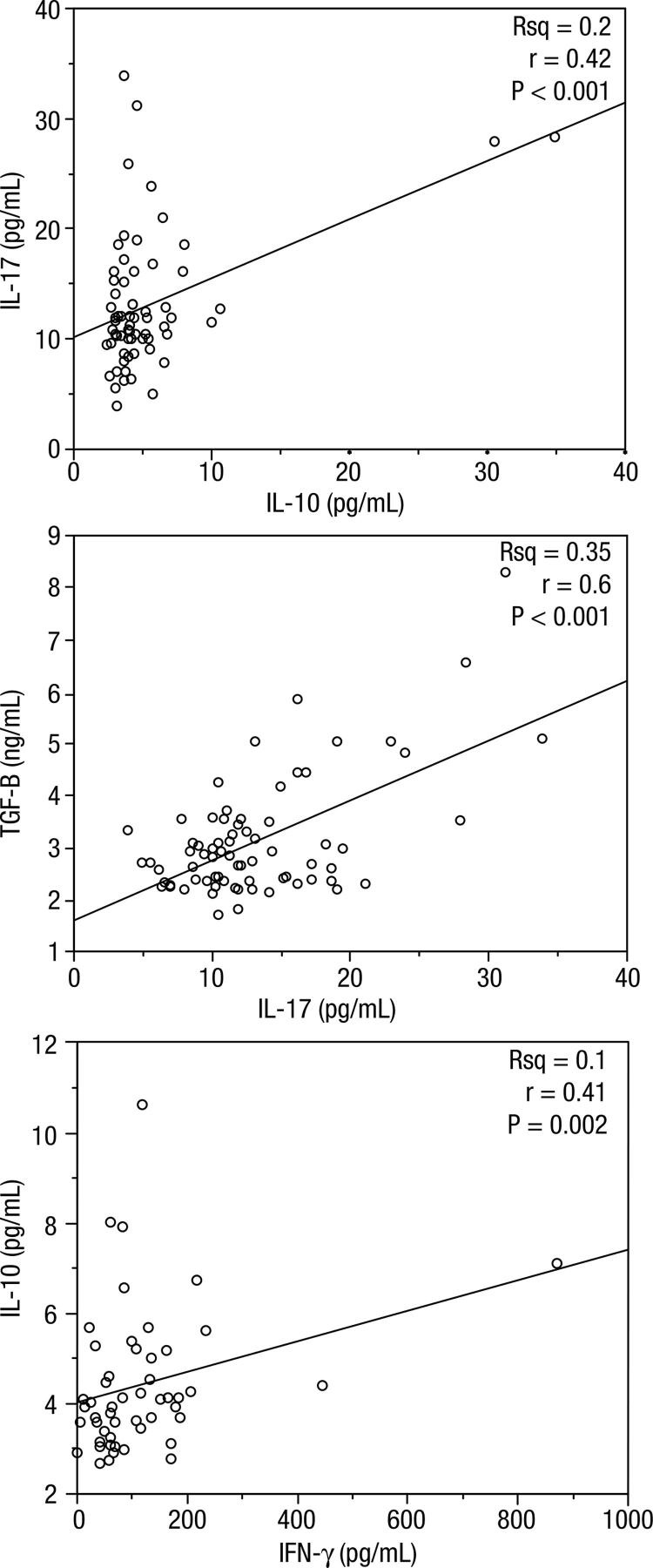
Correlation between baseline concentrations of Th17 and Treg related cytokines in all of participants.

Figure 1 presents the serum Th17 and Treg-related cytokines before and after intervention. Baseline concentrations of IL-6 were significantly higher in obese than non- obese group. However baseline concentrations of IFN-γ and TGF-β were non-significantly higher in obese groups. Vitamin A supplementation significantly reduced serum concentrations of IL-17 and TGF-β in non-obese and obese groups respectively. Serum IL-10 concentrations significantly decreased in non-obese vitamin A treated group and serum IL-6 concentrations after intervention reduced in all three groups. Because of the possible influence of significant difference in initial concentrations of cytokines in obese vitamin A-treated group compared with other groups, we analyzed the relative percentile change from baseline in biochemical parameters in treatment groups; however there was no significant difference between relative percentile changes from baseline in cytokine concentrations between groups (data not shown). Serum IL-17 concentrations were positively correlated with serum IL-10 and TGF-β in total participants (Figure 2). There was also a positive relationship between Serum IL-10 and IFN-γ concentrations in combined analysis of groups. Comparison of serum pro-inflammatory to anti-inflammatory cytokines ratios are presented in table 2. Obese vitamin A – treated group had significantly higher concentrations of IL-6/IL-10 ratio and IL-6/TGF-β ratio compared with other groups (multiple comparisons with Bonferroni corrections). IL-17/IL-10 ratio was higher in obese vitamin A received group which was at the border of statistical significance. We also measured serum Retinol Binding Protein/Transthyretin ratio (RBP/TTR) for evaluation of vitamin A status. After vitamin A supplementation serum RBP/TTR ratio only in non-obese group increased; however this increase was not significant (14). No side effects of vitamin A have been reported in the present study. In basic lab biochemistry tests (fasting blood glucose, lipid profile) and liver enzymes (serum aspartate transaminase and alanine transaminase) only a physiologically mild and clinically insignificant elevation had been observed (13).

**Table 2:** Comparison of baseline ratio of pro-inflammatory to anti-inflammatory cytokines between study groups

	Obese – vitamin A	Obese – placebo	Non-obese	P-value*
n	27	23	25	
IL-6 /IL-10 (ratio)	10.82 ± 6.11	5.18 ± 3.72	7.73 ± 9.23	0.019^†^
IL-6/TGF-β (ratio)	13.87 ± 8.16	8.38 ± 6.76	9.38 ± 9.84	0.011^†^
IL-17/IL-10 (ratio)	3.38 ± 1.48	2.57 ± 1.22	2.90 ± 1.83	0.097
IL-17/TGF-β (ratio)	4.35 ± 1.31	4.14 ± 1.77	4.65 ± 1.97	0.57
IFN-γ/IL-10 (ratio)	27.58 ± 24.73	31.42 ± 27.97	18.49 ± 11.57	0.33
IFN-γ/TGF-β (ratio)	32.38 ± 25.42	51.47 ± 67.71	30.61 ± 18.15	0.25

IL: interleukin; TGF-β: transforming growth factor β; IFN-γ: interferon γ.

* Compared by Kruskal – Wallis test; † obese vitamin A-treated individuals have significantly higher levels than other groups (multiple comparisons with Bonferroni corrections).

## DISCUSSION

Our finding shows for the first time that vitamin A supplementation significantly reduces serum concentrations of TGF-β and IL-17 in obese and non-obese women respectively. Serum levels of IL-10 were also reduced significantly in non-obese women after vitamin A supplementation. IL-17, as a potent mediator of autoimmunity, participates in pro-inflammatory process of several autoimmune disease including rheumatoid arthritis (RA) and multiple sclerosis in humans (MS) and collagen induced arthritis (CIA) and EAE in animals (15). Therefore, therapeutic agents which reduce the IL-17 production might be a good approach to control the autoimmune disease. Schambach and cols. (16) evaluated the effects of all trans retinoic acid (ATRA) on the expression of IL-17 in mice; they showed that ATRA leads to decreased induction of IL-17 expressing T cells through activation of retinoic acid receptor-α (RAR-α). This was in consistent with Elias and cols. (11) findings reported the inhibitory effect of ATRA on Th-17 cell formation.

ATRA as a potent derivate of vitamin A is considered as a therapeutic agent in prevention or treatment of autoimmune disease and several types of cancers (17,18) however its potential adverse effects and life-threatening complications such as headache, nausea, vomiting, irregular heartbeat, bleeding, liver dysfunction and teratogenicity should not be forgotten (19). In the human body, vitamin A as retinyl palmitate is converted to ATRA by dendritic cells (DC) from mesenteric lymph nodes and Peyer’s patches (16) and it capable to suppress carcinogenesis or other degenerative disease with less (20) or no side effects (21,22).

It has been reported that regulatory T cells can increase IL-17 production by means of TGF-β secretion in presence of IL-6 (23,24) and blocking TGF-β signals leads to reduction in IL-17 production (9). Similarly, serum IL-10 reduction was in parallel of IL-17 reduction in non – obese women. There are several contrasting roles for the effect of retinoids on IL-10 production; IL-10 is an immunosuppressive cytokine acts as a signature of T-regulatory cells and its peripheral induction is done in the presence of TGF-β. Consistent with our results, Maynard and cols. (25) reported that inhibitory effect of retinoids on TGF-β mediated IL-10 gene expression by naïve CD4^+^ T cells happens at the level of transcription. In contrast with the Sumarac-Dumanovic and cols. findings (7) we observed no significant difference in serum IL-17 between obese and non-obese groups. However, baseline concentrations of IL-6, another signature cytokine of Th17 cells, were significantly higher in obese compared with non-obese women. Our finding was in consistent of several previous reports (26,27). IL-6 is expressed in both subcutaneous and visceral adipose tissue and in human, circulating amounts of this cytokine is elevated with adiposity (28). Adipose tissue is capable to produce approximately 25% of circulating IL-6 (29). IL-6 is a potent inducer for hepatic synthesis of CRP and other acute phase proteins in obesity and therefore leads to increased risk of cardiovascular disease (30).

We also evaluate the effect of vitamin A on the circulating amounts of other proinflammtory cytokine, IFN-γ, in obese and non-obese women. Baseline concentrations of IFN-γ were not significantly different between obese and non-obese women. Consistent with our results, Sumarac-Dumanovic and cols. (7) and Svec and cols. (31) found no significant difference between baseline concentrations of IFN-γ in obese versus non-obese women; whereas, Pacifico and cols. (32) showed that obesity is associated with increased T-helper IFN-γ secreting cells in obese children. This inconsistency might be explained by age and sex related response of immune cells or it can also occur because of counter- regulatory mechanisms of Th1 versus Th2 immune cell response as revealed by Sumarac-Dumanovic and cols. (7). Our data also indicate that vitamin A had no considerable effect on serum concentrations of IFN-γ in obese or non-obese women. Baseline serum concentrations of IL-10 were positively associated with IL-17 and IFN-γ in total participants. As previously reported by Juge-Aubry and cols. (33) white adipose tissue is a regulated source of IL-10 and its production is up-regulated in adipose tissue of obese individuals (34). IL-10 has anti-inflammatory properties and enhanced its production by adipose tissue in obesity is a defense mechanism of body to counter-regulate the pro-inflammatory actions of several inflammatory cytokines such as TNF-α, IL-6 and IL-8. Therefore, the positive relationship between IL-10 and IL-17 or IFN-γ can partly be explained by this mechanism. Randomized double blinded placebo controlled design of the present study was one of the strengths of the present study. However, small sample size and enrolling only women in the trial were limitations of the study.

In conclusion, our results suggest that vitamin A supplementation might reduce Th-17 and T-reg related cytokines in reproductive age women. Non-significant higher levels of several cytokines identify the possible influence of small sample size of the study on the final results. Therefore, additional studies with larger sample sizes are needed to further confirm or possibly reject these findings.
